# Interleukin-10 plays an early role in generating virus-specific T cell anergy

**DOI:** 10.1186/1471-2172-8-8

**Published:** 2007-06-14

**Authors:** Charles H Maris, Craig P Chappell, Joshy Jacob

**Affiliations:** 1 Sidney Kimmel Comprehensive Cancer Center at The Johns Hopkins University, 1650 Orleans Street CRB-1 424, Baltimore, MD 21231, USA; 2 Department of Microbiology and Immunology and the Emory Vaccine Center, Emory University, 954 N. Gatewood Rd, Atlanta, GA 30329, USA

## Abstract

**Background:**

Infection of mice with the Armstrong strain of lymphocytic choriomeningitis virus (LCMV_ARM_) leads to a robust immune response and efficient viral clearance. This is in contrast to infection with the variant strain LCMV_Clone13_, which causes functional inactivation of effector T cells and viral persistence. The mechanism by which LCMV_Clone13 _suppresses the antiviral immune response and persists in its host is unknown.

**Results:**

Here we demonstrate that infection with LCMV_Clone13_, but not with LCMV_ARM_, resulted in a steady increase in the serum levels of the immuno-inhibitory cytokine, IL-10. Blockade of IL-10 using neutralizing monoclonal antibody injections in LCMV_Clone13_-infected mice led to dramatically enhanced effector T cell responses at 8 days post-infection. Even though IL-10 blockade resulted in decreased viral titers, the generation and maintenance of memory T cells was still compromised. The functional inactivation of CD8^+ ^T cells in IL-10-blocked, chronically infected mice 30 days post-infection was incomplete as potent CTL (cytotoxic T lymphocytes) could be generated by *in vitro *re-stimulation. IL-10 knockout mice showed a similar pattern of antiviral CD8 T cell responses: early antiviral T cells were dramatically increased and viral levels were decreased; however, CD8 T cells in IL-10 knockout mice were also eventually anergized and these mice became persistently infected.

**Conclusion:**

Our data suggest that IL-10 plays an early role in LCMV_Clone13_-induced tolerance, although other factors collaborate with IL-10 to induce virus-specific tolerance.

## Background

The immune system is versatile in its ability to respond specifically to a wide variety of infectious agents. Specific CD4 and CD8 T cell responses combined with robust antibody responses clear invading viruses. While the host efficiently clears most viruses, some have evolved strategies to evade the immune system and establish persistent infections. Viral persistence has been linked to a variety of factors, including cell tropism, quantity and duration of antigen persistence and several molecular viral immune evasion strategies [1-8]. The end result is the incapacitation of the immune response and the development of T cell anergy or tolerance [3,9-12].

Cytokines control many aspects of the immune response, and they affect the balance between the development of immunity and tolerance [9,13]. A robust Th1 response is necessary for the resolution of infection and development of immunity for the majority of viral infections. Interleukin 10 (IL-10), a potent anti-inflammatory cytokine, has been shown to dampen Th1 responses [14,15]. Several groups have shown increased IL-10 levels during a variety of persistent bacterial and parasitic infections, including Candida albicans, Trypanosoma cruzi, and Leishmania major [16-19]. Additionally, viral IL-10 homologues have been identified in the human viruses Epstein-Barr Virus (EBV) and Cytomegalovirus (CMV) that have the ability to persistently infect their hosts, furthering speculation that IL-10 plays a role in sabotaging the adaptive immune response [20-22]. Despite the mounting evidence in other systems, a role for host-produced IL-10 during chronic viral infections has not been carefully studied until recently [23,24].

LCMV_ARM_, a natural mouse pathogen, is a prototypic virus infection model for studying effector and memory T cell formation. Infected mice clear LCMV_ARM _within 8 days, and large numbers of memory CD8^+ ^T cells that confer life-long protective immunity persist in the host. In contrast, infection with LCMV_Clone13_, a two amino acid mutant of LCMV_ARM_, leads to persistent viral infection and T cell anergy: anergic CD8 T cells neither kill virus-infected targets nor produce IFN-γ upon peptide stimulation [10]. The current study was undertaken to ascertain if IL-10 plays a role in establishing viral persistence by inducing T cell anergy in LCMV_Clone13_-infected mice. Here we show increased levels of serum IL-10 in mice infected with LCMV_Clone13_, but not LCMV_ARM_. Blocking IL-10 with a neutralizing monoclonal antibody led to partial restoration of T cell function and lowered viral titers if the antibody was administered during T cell priming. The critical window for IL-10 in establishing anergy is early during the immune response, as blockade of IL-10 after viral persistence is established had no effect. However, the necessity of IL-10 in establishing T cell anergy is not complete in that LCMV_Clone13 _established a persistent infection in IL-10 deficient mice, despite a robust early CD8 T cell response. This report clearly links persistent LCMV infection of mice, and the ensuing T cell anergy, to host-produced IL-10.

## Results

### Increased IL-10 expression following infection with LCMV_Clone13 _but not LCMV_ARM_

**Figure 1:**
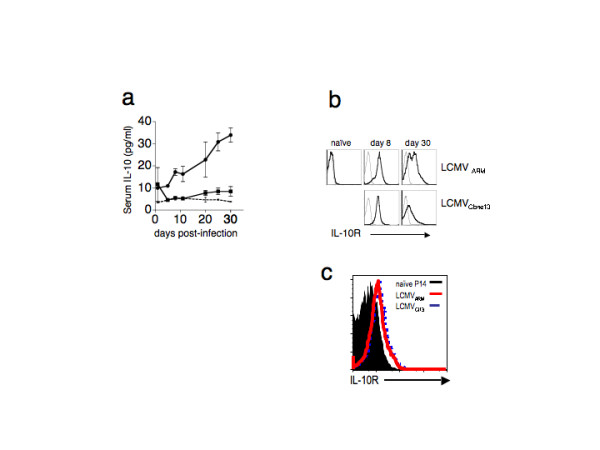
**Serum IL-10 levels in LCMV infected mice**. **a) **Sera from naïve (dashed line) or mice infected with LCMV_ARM _(squares) or LCMV_Clone13 _(circles) were assayed for IL-10 by ELISA at the indicated time points. The limit of detection of IL-10 by ELISA was 4 pg/ml. Serum IL-10 levels in naïve mice were at or below the limit of detection. Each time point represents 3–10 mice. **b) **IL-10 receptor expression was measured on CD8^+ ^T cells in naïve mice and days 8 and 30 post infection by LCMV_ARM _and LCMV_Clone13_. The light line is staining by the isotype control and the bold line is IL-10R expression on gated CD8^+ ^T lymphocytes. **c) **IL-10R expression on virus-specific CD8 T cells is upregulated in the contexts of acute and viral infection. Mice were infected with LCMV_ARM _(bold red line) or LCMV_Clone13 _(dashed blue line) and IL-10R expression was assessed eight days post-infection. Lymphocytes were gated on CD8 and D^b^GP33 tetramers. The solid histogram is the expression of IL-10R on naïve P14 CD8^+ ^lymphocytes.

Increased levels of inhibitory cytokines such as Interleukin 10 (IL-10) have been demonstrated during persistent viral infections in humans [25,26]. Infection of mice with LCMV_Clone13 _induces anergy in CD4 and CD8 T cells, whereas infection with LCMV_ARM _leads to robust immunity [10,27]. We were interested to see if persistent LCMV infection led to increased levels of serum IL-10. We infected cohorts of mice with either LCMV_ARM _or LCMV_Clone13_, collected their sera at various time points post-infection (p.i.) and measured the IL-10 levels by ELISA (Figure 1a). Other than an early spike of IL-10 detectable on day 2 post-infection, LCMV_ARM_-infected mice had low levels of IL-10 in their serum. IL-10 levels in LCMV_ARM_-infected mice increased slightly above naïve mice as immune memory developed beyond day 20. In contrast, LCMV_Clone13_-infected mice accumulated IL-10 in their serum. Beyond day 2 post-infection, LCMV_Clone13_-infected mice had significantly higher levels of serum IL-10 compared to acutely infected mice, and the levels increased with time. The steady increase in serum IL-10 was concomitant with the functional inactivation, i.e. loss of cytolytic activity and IFN-γ production, in CD8 T cells ([28] and Figure 3). These data correlate the T cell anergy observed in persistently LCMV infected mice with an increase in serum IL-10.

CD8 T cells are critical for controlling chronic LCMV infection [27]. In order for IL-10 to directly effect CD8 T cells, they must express the IL-10 receptor. We confirmed IL-10R expression on CD8 T cells during the course of acute and chronic infections. In our hands, IL-10R is universally high at day 8 post-infection for both acute (LCMV_ARM_) and chronic (LCMV_Clone13_) infections. By day 30, IL-10R expression has decreased on a subset of memory CD8 T cells in acutely infected mice. Persistently infected mice, however, lose IL-10R expression on the majority of CD8 T cells, consistent with the deletion of the majority of anti-viral CD8 T cells. We next assessed IL-10R expression directly on LCMV-specific CD8 T cells. We looked at splenic GP33-specific CD8 T cells eight days post-infection, a time during which chronically infected mice contain virus-specific T cells with slightly impaired effector functions. GP33-specific CD8 T cells in both acutely and chronically infected mice had higher IL-10R expression when compared to naïve CD8 T cells. These data are consistent with the model that IL-10 directly affects CD8 T cells.

### IL-10 blockade enhances IFN-γ production in persistently infected mice 8 days post-infection

**Figure 2:**
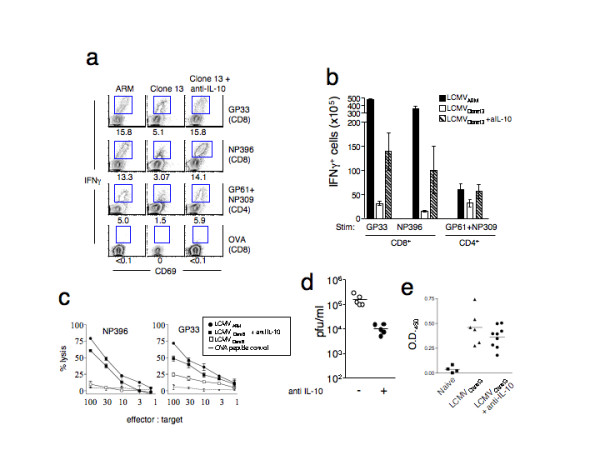
**Neutralizing IL-10 leads to enhanced T cell responses in LCMVClone13-infected mice at day 8 post-infection**. **a) **Splenocytes from mice infected with LCMV_ARM _(left column), LCMV_Clone13 _(middle column), or LCMV_Clone13 _and given anti-IL-10 antibodies (right column), were stimulated with LCMV class I peptides, GP33-41 or NP396-404, class II peptides, GP61-80 and NP309-324 and control ovalbumin (OVA) peptides and IFNγ production was measured by intra cellular cytokine staining. Numbers below representative plots indicate the frequency CD69^+ ^IFNγ^+ ^T cells. The cells gated (CD4 or CD8) are indicated in parentheses. Plots are representative of 5 mice in each group. **b) **The total number LCMV-specific IFN-γ^+ ^splenic CD8 and CD4 T cells from the mice infected with LCMV_Clone13_, or LCMV_Clone13 _treated with anti IL-10 are shown in open and hatched bars, respectively. Data from acutely-infected LCMV_ARM _mice are shown in the filled bars for comparison. The LCMV peptides used for stimulation and the cell gating strategy are indicated below the graph. These data are representative from two independent experiments; the average of 5 mice per group is graphed. **c) **Anti-IL-10 treated, LCMV_Clone13_-infected mice exhibit potent CTL responses. Splenocytes from uninfected or mice were infected with LCMV_ARM_, LCMV_Clone13_, LCMV_Clone13 _plus anti-IL-10 were tested for CTL activity against target cells pulsed with LCMV NP396-404, GP33-41, or control OVA peptides (not shown), 8 days p.i. using a standard 51^Cr ^release assay [33]. **d) **LCMV_Clone13_-infected mice receiving anti-IL-10 mAbs have lower viremia. Day 8 p.i. sera from LCMV_Clone13 _infected mice with (filled circles) or without (open circles) anti-IL-10 treatment were tested for infectious virus by plaque assay [35]). **e) **Anti-IL-10 treatment did not lead to enhanced humoral responses at day 8 post-infection. Sera were collected from mice infected with LCMV_Clone13 _with and without anti-IL-10 treatment at 8 days post-infection and LCMV-specific antibodies were assessed by ELISA. There was no statistical difference (p = 0.2) in the serum anti-LCMV antibody levels between these two groups.

We next wanted to determine if neutralizing IL-10 would have an effect on the developing T cell response during a chronic viral infection. We injected LCMV_Clone13_-infected mice with anti-IL-10 antibodies or normal rat IgG on days 0, 2, and 4 after infection. Eight days following infection, CD8^+ ^and CD4^+ ^T cell responses were measured by peptide-induced IFN-γ production and cytolytic killing of peptide-pulsed target cells. LCMV_Clone13_-infected animals exhibited depressed CD8 T cell responses: the frequency of cells responding to the immunodominant epitopes NP396 and GP33 was reduced by 60–80% when compared to LCMV_ARM_-infected mice (Figure 2a). In contrast, mice that received IL-10 blockade exhibited enhanced T cell responses. The frequencies of NP396 and GP33 tetramer^+ ^CD8 T cells were comparable to those observed in LCMV_ARM_-infected mice (data not shown). The amount of IFN-γ production per cell, as measured by mean fluorescence intensity (MFI) was higher in anti-IL-10 treated mice than in LCMV_Clone13_-infected, rat IgG treated controls for both LCMV peptides, GP33 (350 ± 12.5 vs. 248.5 ± 39.5 for anti-IL-10 treated vs. rat IgG treated) and NP396 (204 ± 30 vs. 116.7 ± 12.2).

LCMV_Clone13_-infected mice receiving anti-IL-10 therapy exhibited a level of IFN-γ production that was indistinguishable from LCMV_ARM_-infected mice (Figure 2a). Mice receiving anti-IL-10 therapy had larger spleens and significantly more (5–7 fold) virus-specific CD8^+ ^T lymphocytes, relative to untreated LCMV_Clone13_-infected control mice (Figure 2b).

CD4 T cells are essential for maintaining antiviral CD8 T cells [27,29]. Therefore, we monitored the effect of anti-IL-10 treatment on rescuing virus-specific CD4 T cell function by testing their ability to produce IFN-γ upon stimulation with the LCMV MHC class II peptides GP61 and NP309. Anti-IL-10-treated mice exhibited higher levels of IFN-γ production by CD4^+ ^T cells at 8 days p.i. (5.5% ± 1.2 vs. 1.5% ± 0.7 for anti-IL-10 treated vs. untreated LCMV_Clone13_-infected mice, p = 0.033, Figure 2a). The absolute numbers of LCMV-specific CD4 T cells were also higher in the anti-IL-10 treated LCMV_Clone13_-infected group as compared to LCMV_Clone13_-infected mice that did not receive anti-IL-10 treatment (Figure 2b). Thus, neutralizing IL-10 during the time that T cell priming occurs drastically enhanced peak antigen-specific CD4 and CD8 T cell responses, both in absolute numbers and in function as measured by IFN-γ production.

### IL-10 blockade restores CTL activity and lowers viral load

Cytolytic killing is a hallmark of activated antigen-specific CD8 T cells, and it is one of the first properties to be lost in persistently infected mice [10,27]. We wanted to know if IL-10 blockade restored the cytolytic activity of CD8 T cells. We measured the cytolytic activity of CD8 T cells by ^51^Cr release assay using target cells pulsed with the immunodominant LCMV peptides NP396 and GP33 (Figure 2c). Killing of target cells was dramatically impaired in LCMV_Clone13_-infected mice at 8 days p.i., but neutralizing IL-10 *in vivo *restored cytotoxic activity nearly to levels observed in LCMV_ARM_-infected mice (Figure 2c). We then tested the mice to determine whether enhanced T cell responses induced by neutralizing IL-10 led to a reduction in viral load. Despite the improved CTL and cytokine responses in mice receiving IL-10 blockade (Figure 2a–c), they still had infectious virus (Figure 2d). However, the level of viremia was significantly reduced (> 1 log) in mice receiving IL-10 blockade (1.6 × 10^5 ^± 3.9 × 10^4 ^pfu/ml, n = 5, for LCMV_Clone13 _infections vs. 2.3 × 10^4 ^± 6.7 × 10^3 ^pfu/ml, n = 5, for LCMV_Clone13 _plus anti-IL-10, p = 0.0076). The lowered viral titers observed at day 8 p.i. could be due to enhanced cytolytic T cell activity or due to an enhanced antibody response to LCMV in anti-IL-10 treated mice, or a collaboration of the two effects. To distinguish between these two possibilities we compared the anti-LCMV antibody responses at day 8 post-infection in groups of LCMV_Clone13_-infected mice with or without anti-IL-10 treatment. Anti-IL-10 blockade did not result in enhanced virus-specific humoral responses (Figure 2e). LCMV-specific IgG levels in the IL-10-blocked, persistently infected mice were not significantly different than untreated controls at day 8 post infection (p = 0.2). The data suggest that enhanced cytotoxic CD8 T cell responses and not humoral responses are responsible for the lowered viral titers observed in the anti-IL-10 treated mice early in the response. These data further support the concept that host-produced IL-10 directly impacts LCMV-specific CD8 T cells early during the immune response.

### Despite the initial enhanced response, mice receiving a short course of IL-10 blockade contain a mixture of functional and anergized CD8 T cells, and exhibit a low-level persistent viremia

**Figure 3:**
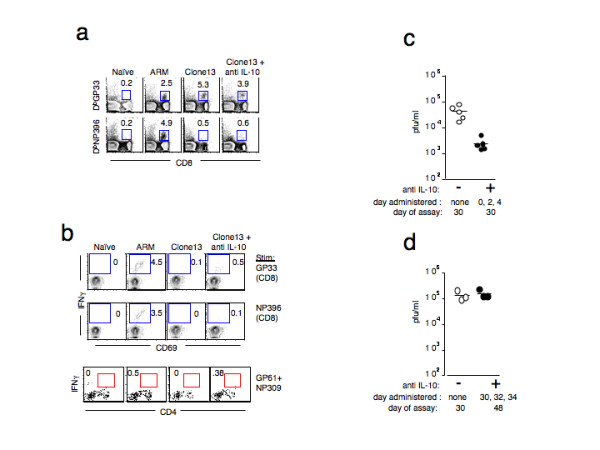
**Early anti-IL-10 therapy improves virus control 30 days p.i**. **a) **Spleen cells from naïve, LCMV_ARM_, and LCMV_Clone13_-infected mice with and without anti-IL-10 treatment (day 30 p.i.) were stained with LCMV tetramers D^b^GP33 and D^b^NP396. The numbers shown are the frequency of tetramer^+ ^cells per CD8^+ ^T cells. Plots are representative of 5 mice. **b) **IFNγ production by CD8 T cells 30 days p.i. Splenocytes were stimulated with LCMV MHC class I restricted peptides, GP33-41 or NP396-404 or class II peptides GP61-80 and NP309-324 and then assayed for IFNγ production by ICCS. All plots are gated on CD8 T cells and the numbers indicate the frequency of CD69^+ ^IFNγ^+ ^cells. **c) **Anti-IL-10 treated mice exhibited lower viremia at 30 days p.i Sera from LCMV_Clone13_-infected mice without (open circles) and with (filled circles) anti-IL-10 treatment were tittered for infectious virus by plaque assay. Naïve and LCMV_ARM _immune mice were free of virus (not shown). **d) **Anti-IL-10 treatment in mice with established persistent infections does not lead to lowering of viral titers. Cohorts of mice infected 30 days earlier with LCMV_Clone13 _were injected with anti IL-10 therapy on days 30–34 p.i Their sera were collected two weeks later and assayed for infectious virus by plaque assay. The LCMV titers from untreated (open cirlces) and anti-IL-10 treated (filled circles) mice are shown.

In LCMV_Clone13_-infected mice receiving IL-10 blockade, IFN-γ production and cytolytic killing by CD8 T cells were intact, and CD4 T cell responses were also enhanced at day 8 p.i. (Figure 2a). It was essential to determine if this potent antiviral immune response could develop into immune memory and resolve the viral infection. We analyzed CD8 T cells from immune LCMV_Clone13_-infected mice (day 30) with and without IL-10 blockade for LCMV-specific MHC/tetramer binding (Fig. 3a) and IFN-γ production (Fig. 3b). In untreated LCMV_Clone13_-infected mice, we observed large numbers of GP33 tetramer^+ ^CD8 T cells that failed to produce IFN-γ, a characteristic phenotype of anergized virus-specific T cells [10]. CD8 T cells specific for the NP396 epitope were mostly deleted and the rare NP396 tetramer binding cells that were present failed to produce IFN-γ upon peptide stimulation. In contrast, mice receiving IL-10 blockade had a small fraction of CD8 T cells that produced IFN-γ when stimulated with GP33 peptide (0.5% ± 0.2 vs. 0.1% ± 0.1%, Fig. 3b), indicating that, at least on some level, functional CD8 T cells were present in persistently infected mice that received IL-10 blockade. Persistently infected, anti-IL-10 treated mice deleted NP396-specific CD8 T cells, similar to LCMV_Clone13_-infected, rat IgG-treated control mice. Taken together, the tetramer and IFN-γ data for two immunodominant epitopes suggest that the generation of virus-specific memory cells was impaired although some GP33-specific CD8 T cells that retain IFN-γ production persisted in mice that received IL-10 blockade (Figure 3b). We also monitored CD4 T cell responsiveness at 30 days p.i. A small population of GP61/NP309-specific CD4^+ ^T cells remained in IL-10 blocked mice, although the MFI of IFN-γ production was low (Figure 3b).

Anti-IL-10 treated, LCMV_Clone13_-infected mice remained persistently infected at 30 days p.i. (Figure 3c). However, viremia was approximately 1.5 logs lower in LCMV_Clone13_-infected mice that received anti-IL-10 therapy (2.4 × 103 ± 6.6 × 102 pfu/ml, n = 5, for LCMV_Clone13 _plus anti-IL-10 vs. 4.2 × 104 ± 1.1 × 104 pfu/ml, n = 5, for LCMV_Clone13_).

### IL-10 blockade in mice with established persistent infections did not result in enhanced T cell responsiveness or lowered viral titers

Blocking IL-10 early in the infection during T cell priming (days 0–4) led to enhanced LCMV-specific T cell responses and lower viral titers. We wanted to know if blocking IL-10 was a useful strategy in lowering viral titers in mice with established persistent infections. We chose mice that were persistently infected with LCMV_Clone13 _(day 30 p.i.) and injected them with normal rat IgG or anti-IL-10 antibodies on days 30, 32 and 34 post-infection. Two weeks following anti-IL-10 treatment we assayed these mice for CD4 and CD8 T cell function and measured serum viral titers. IL-10 blockade after establishment of viral persistence had no effect on LCMV-induced T cell tolerance. T cell anergy could not reversed (data not shown), and there was no change in viremia (Figure 3d). T cells from mice treated with anti-IL-10 late in the infection were non-functional and indistinguishable from untreated LCMV_Clone13_-infected mice (data not shown). These data suggest that there is an early critical window during which IL-10 works to induce anergy in T cells.

### Mice receiving early anti-IL-10 therapy exhibit CTL activity following in vitro re-stimulation

**Figure 4:**
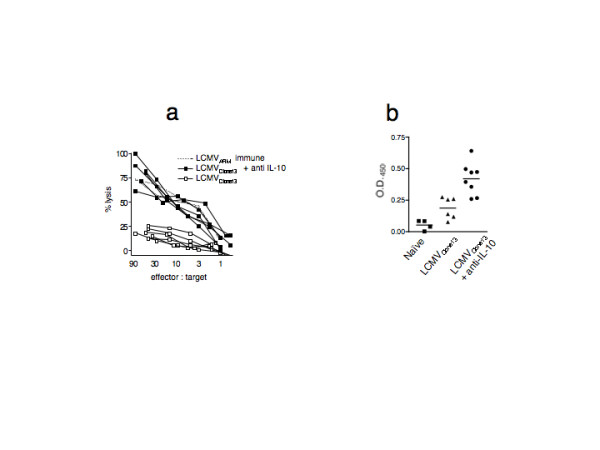
**LCMVClone13 infection induced anergy induced in CD8 T cells is reversed by anti-IL-10 treatment**. **a) **Splenocytes from LCMV_ARM _immune (dashed line), LCMV_Clone13_-infected with (closed square) or without (open square) anti-IL-10 (day 30 p.i.), were stimulated in vitro for 5 days with LCMV GP33 peptide and IL-2, and their capacity to lyse LCMV GP33 peptide-pulsed targets was measured by 51^Cr ^release assay. The effector to target ratio was calculated based on CD8 frequency. There were 5–7 mice per group, each line represents one mouse. Background killing in unpulsed target cells was less than 10%. **b) **The amount of anti-LCMV IgG, 30 day p.i., in the sera of naïve (filled squares), LCMV_Clone13 _infected with (filled triangles) and without anti IL-10 treatment (filled circles) was measured by ELISA. The difference in anti-viral IgG between the LCMV_Clone13_-infected mice receiving anti-IL-10 versus untreated is statistically significant (p = 0.002).

Next, we wanted to know if the anergy observed in anti-IL-10 treated, LCMV_Clone13_-infected mice was complete and irreversible, or if the effector/memory T cells that remained could expand and function. Splenocytes from LCMV_Clone13_-infected mice (day 30 p.i.) were stimulated in vitro with GP33 peptide, in the presence of IL-2, for 5 days and their ability to kill target cells was determined by ^51^Cr release assay (Figure 4a). Re-stimulation *in vitro *restored cytolytic activity of LCMV_Clone13_-infected anti-IL-10 treated mice; their CTL activity was comparable to LCMV_ARM_-immune mice. In contrast, GP33-specific CD8 T cells from LCMV_Clone13_-infected, untreated mice were refractory to peptide re-stimulation and failed to kill target cells. These data clearly demonstrate that IL-10 blockade early in the viral infection led to incomplete tolerization of virus-specific CD8 T cells despite the fact that these mice had low-level viremia.

### Mice receiving anti IL-10 therapy have an enhanced antibody response against LCMV 30 days post-infection

We assessed the antiviral antibody titers 30 days p.i. in LCMV_Clone13_-infected mice with and without anti IL-10 treatment (Figure 4b). Interestingly, mice receiving IL-10 blockade had significantly greater levels of LCMV-specific antibodies than mice that did not receive anti-IL-10 therapy (O.D. 0.4201 ± 0.045, n = 8 vs. 0.1875 +/- 0.035, n = 6, p = 0.002). The level of antiviral IgG in LCMV_ARM _immune mice was much greater than the LCMV_Clone13_-infected mice that received anti-IL-10 blockade (1.151 ± 0.042, n = 10 versus 0.4201 ± 0.045, n = 8, p < 0.0001, not shown). These data, combined with the low antiviral antibody levels seen 8 days p.i., show that the LCMV-specific antibody response matured with time in the IL-10 blocked mice.

### IL-10 knockout mice recapitulate the phenotype observed in anti-IL-10 antibody treated mice: early T cell enhancement followed by induction of anergy

**Figure 5:**
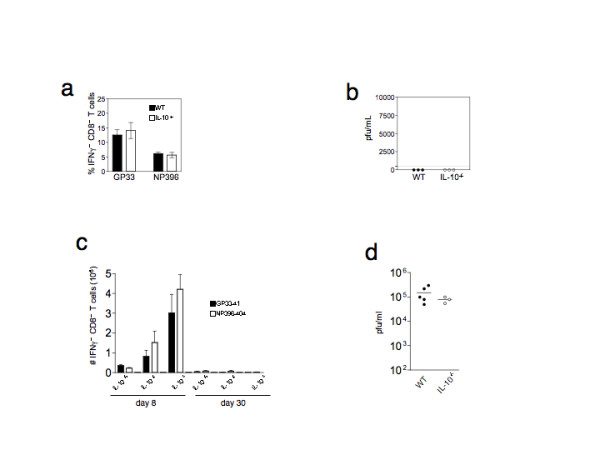
**IL-10-/- efficiently clear LCMVARM but upon infection with LCMVClone13 these mice exhibit an early enhanced T cell response, followed by anergy and viral persistence**. **a) **IL-10^-/- ^(open bars) or control wild type (WT) C57BL/6 (filled bars) mice were infected with LCMV_ARM _and 8 days later the CD8 T cells were assayed for their ability to produce IFN-γ upon stimulation with LCMV peptides, GP33 and NP396. The frequencies ( ± standard deviation) of IFN-g^+ ^CD8^+ ^T cells for the two groups of mice are shown; there was no significant difference between the groups. **b) **IL-10^-/- ^mice clear LCMV_ARM _as efficiently as C57BL/6 mice. Groups of mice were infected with 2 × 105 pfu LCMV_ARM _and eight days post infection, the sera from IL-10^-/- ^(open circles) and WT, C57BL/6 (filled circles) mice were assayed for infectious virus by plaque assay. **c) **Cohorts of C57BL/6 (IL-10^+/+^), IL-10^+/- ^and IL-10^-/- ^mice were infected with LCMV_Clone13 _and 8 or 30 days later, the ability of CD8 T cells to produce IFN-g upon stimulation with LCMV peptides, GP33 (filled bars) and NP396 (open bars) were assayed by intracellular cytokine staining. The absolute numbers of splenic IFN-γ^+ ^CD8^+ ^T cells ( ± SD) for the three groups are plotted. Robust CD8 T cells responses were observed in IL-10^-/- ^mice. For each of the peptide stimulation at day 8, the numbers of IFN-γ^+ ^CD8^+ ^T cells in IL-10^-/- ^were statistically significant than those in IL-10^+/- ^(p < 0.01) and IL-10^+/+ ^(p < 0.001) mice. The numbers of IFN-γ^+ ^CD8 T cells in IL-10^+/- ^and wild type, IL-10^+/+ ^mice were not statistically significant. **d) **Both IL-10^+/+ ^and IL-10^-/- ^mice remained persistently infected, 30 days after infection with LCMV_Clone13_. Sera from infected, IL-10^-/- ^(open circles) and IL-10^+/+ ^(filled circles) were assayed for virus by plaque assay. The difference in the viral titers between the two groups was not statistically significant. Each dot represents an individual mouse.

The data presented so far clearly show that IL-10 blockade led to enhanced early T cell responses but not the eradication of virus. One possibility is that the T cell anergy observed at 30 days p.i. could be due to the inefficiency of the antibody treatment. Continued blockade of IL-10 throughout the course of persistent LCMV_Clone13 _infection may result in complete viral clearance and the development of T cell immunity. To decisively determine the causal role of IL-10 in generating T cell anergy during persistent viral infections, and to avoid the vagaries of multiple injections of heterospecific neutralizing antibodies, we attempted to persistently infect IL-10 deficient mice. We first tested the ability of IL-10^-/- ^mice to clear acute LCMV_ARM _infections. Cohorts of IL-10^-/- ^mice were infected with 2 × 10^5 ^pfu of LCMV_ARM _and their ability to mount T cell responses and clear virus was assayed at 8 days post infection. IL-10^-/- ^mice mounted robust CD8 T cell responses (Figure 5a) and cleared LCMV_ARM _(Figure 5b) as efficiently as wild type mice. We then infected IL-10^-/- ^mice with LCMV_Clone13 _and examined their T cell responses and ability to clear virus at days 8 and 30 p.i. IL-10^-/- ^mice exhibited exaggerated T cell responses at 8 days p.i., while wild type mice exhibited virus-induced immune suppression. Interestingly, heterozygous IL-10^+/- ^littermate controls exhibited T cell responses intermediate between those of control IL-10^+/+ ^and IL-10^-/- ^mice (Figure 5c). However with time, IL-10^-/- ^mice, as well as IL-10 heterozygotes, lost their virus-specific CD8^+ ^T cells and became persistently infected by day 30 p.i. (Figures 5c, d). We also tracked the IFN-γ responses to three other LCMV epitopes, including subdominant epitopes, and the patterns were identical (data not shown); responses to all epitopes tested were absent 30 days p.i. in IL-10^-/- ^mice. These data confirm and extend the observations made using anti-IL-10 antibody treatment and clearly demonstrate that IL-10 plays a direct and early role in generating T cell tolerance; however, additional mechanisms are operating to generate and/or maintain virus-induced anergy.

## Discussion

Early anti-IL-10 therapy resulted in an impressive enhancement in the cellular immune response to LCMV_Clone13 _at 8 days p.i. and the humoral immune response 30 days p.i IL-10-blocked mice had significantly higher IFN-γ production in CD8 and CD4 T cells, both in frequency and magnitude. Cytolytic activity was also enhanced in the anti-IL-10 treated mice. Thus, IL-10 blockade clearly bolstered the early immune response to LCMV such that the immune response in LCMV_Clone13_-infected mice receiving anti-IL-10 was indistinguishable from non-persisting LCMV_ARM _acute infections at 8 days p.i. However, neutralizing IL-10 did not completely block virus-induced CD8 T cell inactivation. Thirty days p.i., LCMV_Clone13_-infected mice receiving anti-IL-10 contained GP33-specific CD8 T cells, the majority of which failed to produce IFN-γ, or were 'exhausted', although, some did retain the ability to produce IFN-γ upon peptide re-stimulation. Interestingly, the functional deficit observed in CD8 T cells in anti-IL-10 treated mice was not complete; cytolytic killing could be rescued by peptide re-stimulation in vitro in the presence of IL-2. While early anti-IL-10 treatment did not prevent viral persistence, these mice exhibited significantly lower viremia compared to rat IgG-treated control mice.

Although IL-10 blocked mice became chronically infected, we were able to rescue cytolytic killing following *in vitro *stimulation in the presence of IL-2 (Figure 4a). However, in our *in vitro *studies it was impossible to determine if we rescued cytolytic killing in all or only a fraction of GP33-specific CD8 T cells. Indirect evidence, presented in Fig. 3a &3b, shows that whereas nearly all virus-specific memory cells in acutely infected mice produce IFN-γ when stimulated, only a fraction of virus-specific CD8 T cells in chronically-infected IL-10-blocked mice were able to make IFN-γ. Although IFN-γ production and IL-2 receptivity are not clearly or directly linked, these data suggest that we were able to rescue cytolytic killing in only a fraction of virus-specific CD8 T cells in the IL-10 blocked mice. Indeed, others have shown that the majority of memory cells proliferate in response to IL-2 [30]. That same group showed that anergized cells did expand in response to IL-2 in the context of persisting antigen, although it was unclear what proportion of virus-specific CD8 T cells were proliferating.

Our data support a scenario in which neutralizing IL-10 enhances the initial CD4 T cells, which in turn leads to an increased magnitude and maintenance of virus-specific CD8 T cells early in the response, possibly by sustained IL-2 production. Additionally, neutralizing IL-10 may have direct effect on CD8 T cells, as indicated by the increased IL-10R expression on virus-specific CD8 T cells (Fig. 1b). Virus-specific CD8 T cells with enhanced cytolytic activity keep the initial virus levels low. At this early phase, antibody levels may play a minimal role. Thirty days later, when the majority of CD8 T cells are rendered non-functional by the persistent virus, viremia is kept low by virus-specific antibodies. It is clear from our data that IL-10 plays a critical role in inducing T cell anergy during persistent LCMV_Clone13 _infections, and that its effect is most potent early in the immune response. Neutralizing IL-10 does not completely block immune tolerance or prevent viral persistence, suggesting that other factors are playing a significant role.

A potential criticism of the antibody-mediated IL-10 neutralization studies is that IL-10 was not blocked throughout the course of the virus-infection. Perhaps continued IL-10 blockade would have led to eventual clearance of virus. Our experiments in IL-10^-/- ^mice unequivocally settle this issue. IL-10 deficiency resulted in improved early adaptive immune responses similar to the antibody-mediated IL-10 neutralization studies. However, with time, CD8 T cells from IL-10^-/- ^mice became non-functional and the mice were persistently infected. Thus, whether by antibody- mediated neutralization or by genetic disruption, the removal of IL-10 had a drastic stimulatory effect on the early antiviral CD8^+ ^and CD4^+ ^T cell responses but its effects were minimal later in the response. This finding leads us to conclude that IL-10 exerts its strongest effect early during infection and other factors operate later during infection to bring about virus-induced T cell tolerance, such as TGF-β or PD-1 expression on CD8 T cells [31].

Inducing IL-10 production in the host or encoding homologues of IL-10 is a common strategy utilized by various pathogens to induce anergy in the host [15]. Here we show that LCMV_Clone13 _also uses this strategy to subvert the host immune response. LCMV_Clone13 _differs from the Armstrong strain by two amino acids, one in the polymerase and the other in the glycoprotein (GP). The mutation in the polymerase enables the virus to replicate at a higher rate than LCMV_ARM _[27,32]. Interestingly, the mutation in GP alters its tropism. Oldstone and colleagues have elegantly shown that LCMV_Clone13 _infects DEC205^+ ^CD11c^+ ^dendritic cells while LCMV_ARM _fails to do so [1,2]. We speculate that infection of DCs with LCMV_Clone13 _leads to IL-10 production by these cells directly or, alternatively, infected DCs could induce regulatory T cells that in turn would secrete IL-10. Our experiments clearly show that while IL-10 is important for virus-induced tolerance induction, there must be other factor(s) that are involved as well. Whether these are IL-10 family members [15] such as IL-19, IL-20, IL-22, AK155 and mda-7 or immuno-modulatory cytokines such as TGF-β, remains to be investigated. Clearly, ligation of PD-1 on CD8 T cells by PD-L1 and/or PD-L2 can have a suppressive effect, but the kinetics of co-stimulation and co-inhibition during chronic LCMV_Clone13 _infections have not been clearly defined.

During the preparation of this manuscript, it has come to the attention of the authors that other laboratories have performed similar experiments with differing results ([24,23]). Although the authors used a blocking antibody to IL-10R that may have been more efficacious then neutralizing IL-10 directly with different kinetics of administration, Brooks *et al*. also performed LCMV_Clone13 _infections in IL-10 knockout mice and observed viral clearance. The route and amount of virus, as well as the genetic background of the IL-10^-/- ^mice, were reportedly identical to those used in our studies; as such, we have no explanation for the difference in outcome.

## Conclusion

The data presented in this paper show that IL-10 assists in generating T cell anergy to a viral infection, and that its role is perhaps most important during T cell priming. Serum IL-10 levels increased during chronic LCMV infections, and neutralizing IL-10 with a monoclonal antibody resulted in improved antiviral CD8 and CD4 T cell responses, as well as antiviral B cell responses. In agreement with our antibody studies, chronically infected IL-10 deficient mice displayed CD8 T cell responses that were indistinguishable from IL-10-blocked mice: the early antiviral response was significantly enhanced. However, both IL-10-blocked mice and IL-10 deficient mice were eventually tolerized to LCMV, evidenced by the presence of anergic virus-specific CD8 T cells and persisting virus. These data suggest that although host-produced IL-10 plays a role in generating virus-specific tolerance early during infection, other factors collaborate to completely tolerize CD8 T cells.

## Methods

### Mice and viral infections

All animal experiments were conducted with IACUC approval. C57BL/6J mice and P14 (B6;D2-Tg(TcrLCMV)327Sdz/JDvsJ) mice maintained on a B6 background were purchased from the Jackson Laboratory (Bar Harbor, ME) and maintained under specific-pathogen-free conditions in the rodent vivarium at the Emory Vaccine Center. IL-10 knockout mice were a kind gift from Dr. Elizabeth Bonney (University of Vermont). Viruses, LCMV_ARM _and LCMV_Clone13_were kind gifts from Dr. R. Ahmed (Emory University) and Dr. A. Zajac (University of Alabama, Birmingham). Non-persistent and persistent infections were generated by injecting mice with 2 × 10^5 ^pfu LCMV_ARM _intraperitoneally or 2 × 10^6 ^pfu LCMV_Clone13 _intravenously, respectively. Cohorts of LCMV_Clone13_-infected mice received normal rat IgG (Sigma) or anti-IL-10 (BD Biosciences) injections either early (200 μg on day 0, 100 μg on days 2 and 4), or late (100 μg each on days 30, 32, and 34).

### Flow cytometry

Flow cytometry was performed on spleen cell suspensions as described [33]. Intracellular cytokine staining (ICCS) was done on splenocytes that were stimulated with LCMV-specific MHC class I peptides, NP396-404 and GP33-41, or MHC class II peptides, NP309-324 and GP61-80 [34], or ovalbumin SIINFEKL (OVA). Anti-CD210 (IL-10R, BD Biosciences) staining was performed intracellularly on peptide stimulated splenocyte cultures.

### IL-10 ELISA

Mouse sera were collected and ELISAs were performed using the IL-10 Quantikine M kit (R & D Systems).

### Viral load plaque assay

Serum LCMV titers in serum were determined by plaque assay as described [33].

### CTL assay

CTL assays were performed using a standard 51^Cr ^release assay using targets pulsed with LCMV NP396-404, GP33-41, or OVA peptides as described [33]. Supernatants from the 51^Cr ^release assay were collected and transferred to Wallac plates, air dried, and read in a Wallac MicroBeta TriLux Liquid Scintillation Counter (Wallac, Turku, Finland). For restimulation experiments, single cell suspensions of spleens from LCMV_ARM _immune mice (n = 2) or LCMV_Clone13_-infected mice with (n = 5, 200 μg anti-IL-10 given on day 0, 2, & 4) or without IL-10 blockade (n = 7) were cultured for 5 days in the presence of 100 U/mL recombinant human IL-2 (R&D Systems). Media was refreshed either other day, no exogenous antigen or peptide was added. After the stimulation, 5 × 105 cells were plated with serial dilutions of 51^Cr^-labeled GP33 peptide-pulsed MC57 g fibroblast targets. Aliquots of stimulated cells were checked by flow cytometry in order to determine the proportion of CD8^+ ^T cells in each sample and the ratio of CD8 T cells : target cells was mathematically adjusted. Percent specific killing was calculated as [(experimental - spontaneous)/(max - spontaneous)] × 100.

### Determination of serum anti-LCMV antibody titer

LCMV-specific serum antibody levels were measured by ELISA as described [35]. Briefly, cell lysates from LCMV infected BHK-21 cells were adsorbed on to Immunosorp (Nunc) plates by overnight incubation at 4°C. Plates were blocked with 4% milk in PBS and serum samples (1:50 dilution) were added and incubated 90 minutes. Following incubation, the plates were washed extensively and the amount of bound IgG was determined by using goat anti-mouse IgG mAb conjugated to horseradish peroxidase (Vector). Bound mouse IgG was visualized by addition of colorimetric peroxidase substrate from R&D Systems.

### Statistical analyses

We used Student's t test analyses to compare the means of different groups of mice. For statistical analysis of data from the IL-10^-/- ^mice, we used one-way ANOVA followed by Bonferroni post test.

## Authors' contributions

CHM conceived and designed the study. CHM and CPC carried out all experiments. JJ participated in the design of the study and assisted CHM in writing the manuscript.
